# Toll-Like Receptor Family Polymorphisms Are Associated with Primary Renal Diseases but Not with Renal Outcomes Following Kidney Transplantation

**DOI:** 10.1371/journal.pone.0139769

**Published:** 2015-10-07

**Authors:** Mark C. Dessing, Jesper Kers, Jeffrey Damman, Henri G. D. Leuvenink, Harry van Goor, Jan-Luuk Hillebrands, Bouke G. Hepkema, Harold Snieder, Jacob van den Born, Martin H. de Borst, Stephan J. L. Bakker, Gerjan J. Navis, Rutger J. Ploeg, Sandrine Florquin, Marc Seelen, Jaklien C. Leemans

**Affiliations:** 1 Department of Pathology, Academic Medical Center, University of Amsterdam, Amsterdam, the Netherlands; 2 Department of Stem Cell Biology and Regenerative Medicine, Keck School of Medicine of the University of Southern California, Los Angeles, California, United States of America; 3 Department of Surgery, University of Groningen, University Medical Center Groningen, Groningen, the Netherlands; 4 Department of Pathology and Medical Biology, University of Groningen, University Medical Center Groningen, Groningen, the Netherlands; 5 Department of Laboratory Medicine, University of Groningen, University Medical Center Groningen, Groningen, the Netherlands; 6 Department of Epidemiology, Unit of Genetic Epidemiology & Bioinformatics, University of Groningen, University Medical Center Groningen, Groningen, the Netherlands; 7 Department of Internal Medicine, Division of Nephrology, University of Groningen, University Medical Center Groningen, Groningen, the Netherlands; 8 Nuffield Department of Surgical Sciences, University of Oxford, Oxford, United Kingdom; 9 Department of Pathology, Radboud University Nijmegen Medical Center, Nijmegen, the Netherlands; University of London, St George's, UNITED KINGDOM

## Abstract

Toll-like receptors (TLRs) play a crucial role in innate- and adaptive immunity. The TLR pathways were shown to play key functional roles in experimental acute and chronic kidney injury, including the allo-immune response after experimental renal transplantation. Data about the precise impact of TLRs and their negative regulators on human renal transplant outcomes however are limited and contradictory. We studied twelve non-synonymous single nucleotide polymorphisms (SNPs) of which eleven in TLR1-8 and one in SIGIRR in a final cohort comprising 1116 matching donors and recipients. TLR3 p.Leu412Phe and SIGIRR p.Gln312Arg significantly deviated from Hardy-Weinberg equilibrium and were excluded. The frequency distribution of the minor alleles of the remaining 10 TLR variants were compared between patients with end-stage renal disease (recipients) and controls (kidney donors) in a case-control study. Secondly, the associations between the minor allele frequency of the TLR variants and delayed graft function, biopsy-proven acute rejection and death-censored graft failure after transplantation were investigated with Cox regression. Carrier frequencies of the minor alleles of TLR1 p.His305Leu (OR = 4.79, 95% CI = 2.35–9.75, *P* = 0.0002), TLR1 p.Asn248Ser (OR = 1.26, 95% CI = 1.07–1.47, *P* = 0.04) and TLR8 p.Met1Val (OR = 1.37, 95% CI = 1.14–1.64, *P* = 0.008) were significantly higher in patients with ESRD, with little specificity for the underlying renal disease entity (adjusted for age, gender and donor-recipient relatedness). The minor allele frequency of none of the TLR variants significantly associated with the surrogate and definite outcomes, even when multivariable models were created that could account for TLR gene redundancy. In conclusion, genetic variants in TLR genes were associated with the prevalence of ESRD but not renal transplant outcomes. Therefore, our data suggests that specific TLR signaling routes might play a role in the final common pathway of primary renal injury. A role for TLR signaling in the context of renal transplantation is probably limited.

## Introduction

Toll-like receptors (TLRs) are pattern recognition receptors (PRR), which can be activated by both pathogen-associated molecular patterns (PAMPs) and endogenous ligands called damage-associated molecular patterns (DAMPs) leading to the induction of an inflammatory response [1, 2]. Single Ig IL-1-related receptor (SIGIRR) is one of the negative regulators of the TLR signalling pathway and is involved in reducing inflammation upon TLR activation to prevent excessive inflammation [3, 4]. TLRs play a part in both the innate and the subsequent adaptive immunity and are of special interest in renal diseases; TLRs are expressed on murine and human leukocytes and renal endothelial and epithelial cells, including podocytes [1, 2]. TLRs are crucial in the antibacterial defence mechanisms during renal infection, however this immune response is detrimental during a sterile inflammatory response including acute and chronic kidney injury and the allo-immune response after transplantation [2, 5]. In renal transplant patients, TLR4 is the most frequently studied TLR family member that is activated by DAMPs that are released during an episode of renal injury and in particular during ischemia-reperfusion injury after long-term cold storage of the transplants. One of the most well-known DAMPs recognized by TLR4 is High-mobility group protein B1 (HMGB1), which is highly expressed in renal transplants of deceased but not living donors after surgery [6]. Different studies have shown that if the donor or recipient inherits or possess a TLR4 loss-of-function single nucleotide polymorphisms (SNPs) such as p.Asp299Gly allele A/G and p.Thr399Ile allele C/T, recipients were less likely to experience delayed graft function (DGF) or acute rejection (AR) [7, 8]. Importantly, recipients with these particular TLR4 variants experienced more episodes of infections [7] highlighting a possible double-edge sword for TLRs in the context of transplantation. Unfortunately, there are conflicting data on the role of TLR4 and other TLR signalling sequence variants on renal outcome in renal transplant recipients [7–18]. This might be explained by the variety in the patient databases that have been used. In addition, studies vary in their definition of study endpoints or studies use only one single endpoint [7–18]. Large cohorts that are adequately powered to investigate the impact of especially multiple SNPs are needed since these pattern recognition receptors are known to be redundant. The aim of the current study is therefore to investigate the impact of SNPs in genes that are involved in TLR pathways on outcomes in the context of renal transplantation.

## Material and Methods

### Study population

Samples were included from a study cohort as described before [19,20]. Between March 1993 and February 2008, 1271 matching donor and recipient peripheral blood mononuclear cells (PBMCs) were obtained from patients who underwent kidney transplantation at the University Medical Center Groningen, The Netherlands. The exclusion criteria were: cases of re-transplantation, combined kidney/pancreas or kidney/liver transplantation, technical problems during surgery, the unavailability of DNA and loss to follow-up. The institutional ethical review board of the University Medical Center Groningen approved the study (METc 2014/077). Written informed consent was obtained from all patients. None of the living transplant donors were from a vulnerable population and all living donors provided written informed consent. In case of deceased donation, the donors provided informed consent when they registered their donation status and by law, no additional consent was needed. The study was conducted according to the principles of the declaration of Helsinki. The final statistical analyses were performed on 1116 individuals (2232 samples), corresponding to 92% of the donor and recipient pairs after exclusion of patients with primary non-functioning grafts (see below).

### DNA isolation, quality control and SNP selection criteria

DNA samples were analyzed for absorbance at 260 nm with NanoDrop spectrophotometer (ND–1000, NanoDrop Technologies) and DNA concentration was calculated by the NanoDrop nucleic acid application module. As a measure of DNA purity 260/280 and 260/230 absorbance ratios were assessed. Where samples failed to meet the minimum DNA concentration and purity recommended for Illumina genotyping, repeated isolation attempts were made. In this study, 12 non-synonymous SNPs in TLR and SIGIRR genes obtained from NCBI (inclusion criterion: minor allele frequency >1%) were analyzed for their association with the various renal outcomes as defined below. Genotyping of the selected SNP was performed using the Illumina VeraCode GoldenGate Assay kit (Illumina, San Diego, CA, USA) according to the manufacturer’s instructions. Genotype clustering and calling was performed using Beadstudio Software (Illumina). Of the chosen SNPs, rs4986790 (TLR4 p.Asp299Gly) and rs4986791 (TLR4 p.Thr399Ile) are in linkage disequilibrium (LD) (r^2^ = 1, SNP Annotation and Proxy Search, Broad institute).

### Case-control study for end-stage renal disease

We performed a case-control study to investigate the association between the TLR SNPs and the prevalence of end-stage renal disease by comparing the recipients as cases with the donors as their respective controls. Cases were separately evaluated for the association of the allele frequencies of the variants in specific groups of renal diseases, namely congenital nephropathy (N = 216), immunecomplex-mediated glomerulonephritis (N = 299), infective pyelonephritis (N = 126) and renovascular disease (N = 102) with the full group of controls (donors, N = 1116). We took into account that donors and recipients are related in case of living related renal transplantation.

### Study endpoints for longitudinal analyses after transplantation

The endpoints used in longitudinal analyses were: delayed graft function (DGF), defined as the requirement for dialysis within the first week after transplantation due to the need for additional renal replacement therapy (patients with subsequent non-functioning and loss of their allograft, referred to as primary non-function (PNF, N = 60) [19], were excluded from analyses), time to the first episode of biopsy-proven acute rejection (BPAR) and death-censored graft failure (defined as the need for dialysis or re-transplantation, patients with PNF were excluded). Data on rejection type (antibody- or T cell-mediated rejection) was unavailable due to the lack of a standardized method for the determination of donor-specific antibodies over time.

### Statistical analyses

Statistical analyses were performed using the R platform for statistical computing version 3.1.1. (www.r-project.org) and PLINK version 1.07 for Mac OS X 10.10.4 (S. Purcell, http://pngu.mgh.harvard.edu/purcell/plink) [21]. We followed the protocol for statistical analyses in genetic studies as described by Clarke *et al*. [22]. Two-sided *P*-values <0.05 were considered statistically significant after Bonferroni correction for multiple comparisons. Minor allele frequencies were calculated as the sum of the minor alleles divided by 2 times the total number of patients. Hardy-Weinberg equilibria for the variants in the donors (as a healthy control group) were calculated and when SNPs were in disequilibrium (after Bonferroni correction), they were excluded from further analyses. In the case-control study, odds ratios and corresponding 95% confidence intervals were calculated with additive genetic logistic regression models correcting for age and gender. Donor-recipient relatedness in case of living related renal transplantation was taken into consideration by applying the DFAM algorithm in PLINK. Because we had a large database with a fixed amount of patients before genotyping started, type II error percentages (100%—power) for the significantly associated variants were calculated *post-hoc* according to the statistical methods as described by Skol *et al*. [23]. The prevalence of end-stage renal disease in The Netherlands was estimated at 0.1% based on the number of patient on renal replacement therapy at time of analysis. The association between single TLR variants and delayed graft function was calculated with univariable logistic regression models and the odds ratios with corresponding 95% confidence intervals were presented. The association between single TLR variants and biopsy-proven acute rejection and death-censored graft failure was calculated with univariable Cox regression models and the hazard ratios with corresponding 95% confidence intervals were presented. Corresponding *P*-values were calculated with log rank tests. In the regression models, all *P*-values underwent Bonferroni correction for multiple comparisons. For the 3 endpoints, we next constructed multivariable models per endpoint that included parameters that are known to influence these endpoints (e.g. cold ischemia time for delayed graft function) and compared these clinical models to models that additionally included all TLR variants in both donors and recipients in order to account for gene redundancy. We used the Akaike information criterion (AIC) to compare the relative goodness-of-fit of the models for their association with the endpoints. Lower AIC values are indicative of a better goodness-of-fit of the model. In this way we wanted to investigate whether including all TLR variants in one multivariable model as a method to account for redundancy between TLRs genes, might provide additional information besides parameters that have been known to influence the outcomes. In this case, the role of all TLR variants *as a group* rather than the possible role for *a single variant* is investigated.

## Results

### Study characteristics and distribution of the TLR gene variants

**Fig 1:**
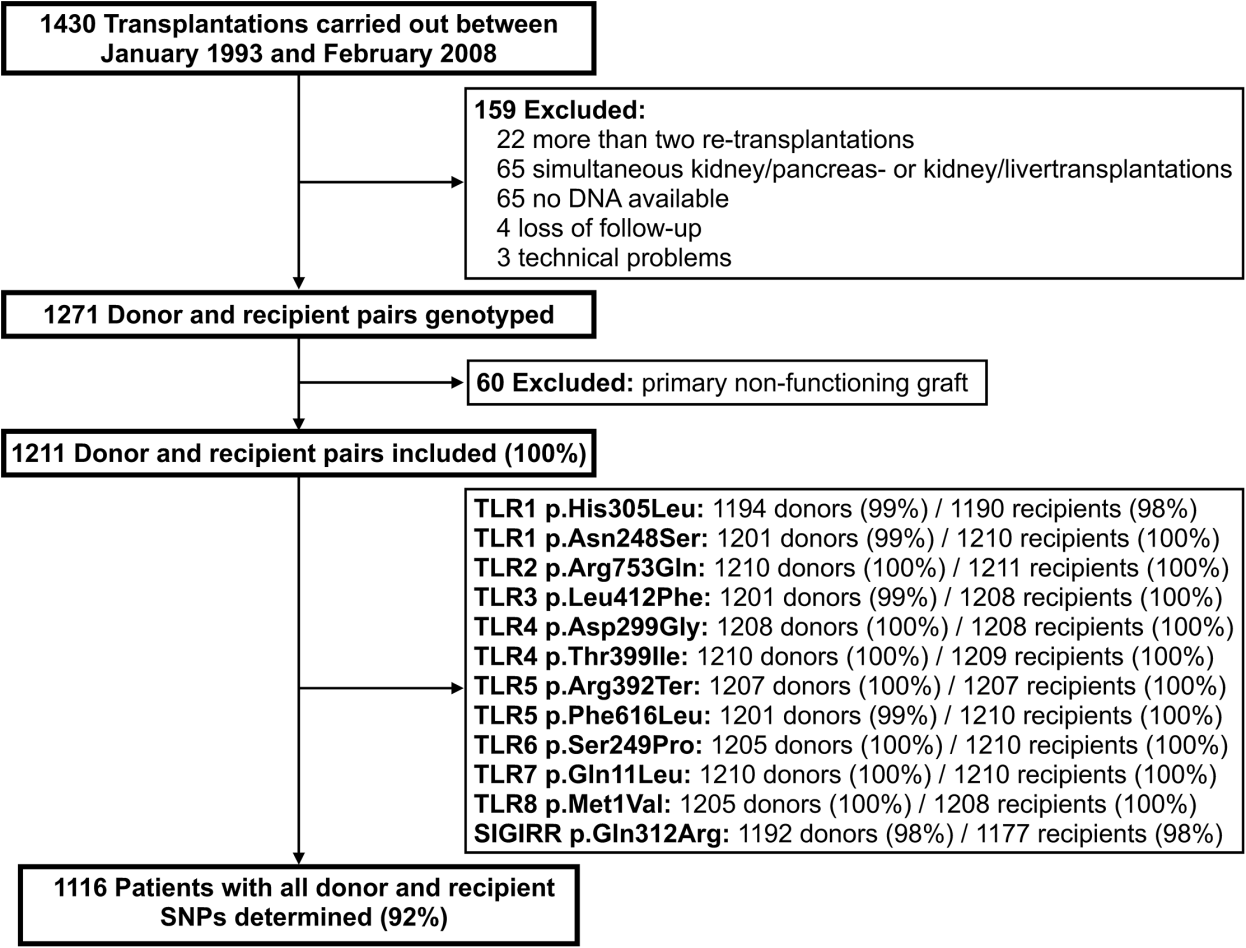
Flowchart of the in- and excluded samples.

Fig 1 shows the flowchart of the in- and excluded patients and samples. The characteristics of cohort are presented in Table 1. There were no significant differences in donor, recipient and transplant characteristics between the included and excluded patient cohorts, which supports our statement that inclusion was not systematically biased. The genotypic distributions of the SNPs in donors and recipients are displayed in Table 2. Of the total of 1211 patients included in the study, 1116 (92%) had all donor and recipient SNPs determined. After Bonferroni correction for multiple testing, TLR3 p.Leu412Phe (*P* = 0.046) and SIGIRR p.Gln312Arg (*P* < 0.0001) appeared in Hardy-Weinberg disequilibrium in controls (donors) and these SNPs were therefore excluded from further analysis.

**Table 1:** Characteristics of the study group, subdivided by included and excluded patients.

Variable	TotalN = 1271	IncludedN = 1116	ExcludedN = 155	P-value^1^
**Donor characteristics**				
Age (mean years ± SD)	44 ± 14	45 ± 14	44 ± 16	1
Male N (%)	645 (51%)	554 (50%)	91 (59%)	0.7
Donor type N (%)				
Living donor	282 (22%)	249 (22%)	33 (21%)	1
Deceased donor (DBD + DCD)	989 (78%)	867 (78%)	122 (79%)	
**Recipient characteristics**				
Age (mean years ± SD)	48 ± 13	48 ± 13	48 ± 13	1
Male N (%)	739 (58%)	655 (59%)	84 (54%)	1
Initial immunosuppression N (%)				
Corticosteroids	1201 (95%)	1053 (94%)	148 (95%)	1
Calcineurin inhibitor (CsA or TAC)	1182 (93%)	1039 (93%)	143 (92%)	1
Proliferation inhibitor (MPA or AZA)	979 (77%)	863 (77%)	116 (75%)	1
mTOR inhibitor	38 (3%)	31 (3%)	7 (5%)	1
Induction therapy N (%)				
Anti-thymocyte globulin	103 (8%)	90 (8%)	13 (8%)	1
Anti-CD3 moab	19 (2%)	15 (1%)	4 (3%)	1
Interleukin–2 receptor antagonist	199 (16%)	171 (15%)	28 (18%)	1
First transplant N (%)	1143 (90%)	1001 (90%)	142 (92%)	1
**Transplant characteristics**				
Cold ischemia time (mean hours ± SD)				
Living donors	2.7 ± 1.9	2.7 ± 2.0	2.6 ± 0.7	1
Deceased donors	20.7 ± 6.5	20.6 ± 6.4	21.6 ± 6.8	1
HLA no. of 0 mismatches N (%)^2^	241 (23%)	213 (23%)	28 (22%)	1

DBD = deceased brain death, DCD = deceased cardiac death, SD = standard deviation, CsA = cyclosporine A, TAC = tacrolimus, MPA = mycophenolic acid, AZA = azathioprine, mTOR = mammalian target of rapamycin, moab = monoclonal antibody, HLA = human leukocyte antigen. HLA = Human leukocyte antigen.

^1^Bonferroni corrected for multiple testing

^2^Data for N = 221 were missing; N = 195 (20%) in the included patients, N = 26 in the excluded (17%), *P* = 0.9.

**Table 2:** Allele frequency distributions and possible phenotypical consequences of the single nucleotide polymorphisms in TLR-related genes.

Gene	Chr	HGVS name (rs number)	Phenotype (refs)	A/a	1000Genomes library				Donor				Recipient			
					A/A (%)	A/a (%)	a/a (%)	MAF (%)	A/A (%)	A/a (%)	a/a (%)	MAF (%)	A/A (%)	A/a (%)	a/a (%)	MAF (%)
TLR1	4	p.His305Leu (rs3923647)	GOF [24,25] LOF [26]	T/a	95.4	4.2	0.4	**2.5**	95.1	4.7	0.2	**2.6**	84.3	11.6	4.1	**9.5**
TLR1	4	p.Asn248Ser (rs4833095)	LOF [25–27]	C/t	53.7	36.6	9.7	**28.0**	54.3	38.1	7.6	**25.9**	50.3	39.0	10.7	**30.0**
TLR2	4	p.Arg753Gln (rs5743708)	LOF [28–30]	G/a	95.2	4.8	0	**2.4**	89.7	10.1	0.2	**5.1**	80.8	19.1	0.1	**9.6**
TLR3	4	p.Leu412Phe (rs3775291)	LOF [31,32] GOF [33]	C/t	44.7	45.7	9.5	**32.4**	49.0	44.6	6.4	**28.7**	52.3	41.2	6.5	**27.1**
TLR4	9	p.Asp299Gly (rs4986790)	LOF [34,35]	A/g	89.3	10.1	0.6	**5.7**	89.3	10.6	0.2	**5.4**	88.7	11.0	0.2	**5.8**
TLR4	9	p.Thr399Ile (rs4986791)	LOF [34,35]	C/t	89.1	10.3	0.1	**5.3**	89.1	10.9	0.1	**5.5**	88.3	11.5	0.2	**6.0**
TLR5	1	p.Arg392Ter (rs5744168)	LOF [36–38]	G/a	88.1	11.7	0.2	**6.1**	86.5	13.4	0.1	**7.1**	84.2	15.2	0.6	**8.2**
TLR5	1	p.Phe616Leu (rs5744174)	GOF [39–41]	A/g	34.8	48.5	16.7	**41.0**	30.6	51.0	18.4	**42.6**	32.4	49.8	17.8	**44.6**
TLR6	4	p.Ser249Pro (rs5743810)	GOF [24]	G/a	35.8	46.7	17.5	**40.9**	35.3	48.0	16.7	**41.0**	39.5	44.0	16.5	**38.6**
TLR7	X	p.Gln11Leu (rs17900)	LOF [42,43]	A/t	71.7	23.3	5.0	**16.7**	68.7	20.1	11.2	**21.1**	71.3	15.9	12.8	**21.7**
TLR8	X	p.Met1Val(rs3764880)	GOF [44,45]	A/g	66.4	15.0	18.6	**26.1**	68.9	18.6	12.5	**22.6**	63.7	16.7	19.6	**27.9**
SIGIRR	11	p.Gln312Arg (rs3210908)	ND	C/t	60.8	34.2	5.0	**22.1**	51.2	47.0	1.8	**25.4**	38.3	49.2	12.5	**37.1**

Donor and recipient genotype are displayed as dominant (A/A), heterozygous (A/a) or recessive (a/a). Chr = chromosome, HGVS = Human Genome Variation Society LOF = loss of function, GOF = gain of function, ND = not determined, MAF = minor allele frequency.

### Frequency distribution of the variant alleles comparing patients with end-stage renal disease and healthy controls

We wanted to find out whether there was a difference in the carrier distribution of the minor allele alleles between patients with end-stage renal disease (recipients) and healthy controls (donors) in a case-control approach. After correction for multiple comparisons, age, gender and donor-recipient relatedness, the carrier frequency of the minor allele for TLR1 p.His305Leu (OR = 4.79, 95% CI = 2.35–9.75, *P* = 0.0002), TLR1 p.Asn248Ser (OR = 1.26, 95% CI = 1.07–1.47, *P* = 0.04) and TLR8 p.Met1Val (OR = 1.37, 95% CI = 1.14–1.64, *P* = 0.008) was significantly higher in patients with end-stage renal disease (Table 3). In Table 4, we separated the patients by cause of end-stage renal disease in order to investigate whether the carrier frequency of the minor alleles for the TLR variants showed specificity for certain underlying disease entities. The minor allele for TLR1 p.His305Leu showed the strongest association with each of the underlying renal disease group tested: congenital nephropathy (OR = 4.94, 95% CI = 2.26–10.84, *P* = 0.0002), immunecomplex-mediated glomerulonephritis (OR = 4.91, 95% CI = 2.31–10.41, *P* = 0.0001), infective pyelonephritis (OR = 6.71, 95% CI = 3.10–14.53, *P* < 0.0001) and renovascular disease (OR = 4.37, 95% CI = 1.83–10.43, *P* = 0.003). TLR1 p.Asn248Ser and TLR8 p.Met1Val showed some specificity for immunecomplex-mediated glomerulonephritis (respectively OR = 1.33, 95% CI = 1.05–1.67, *P* = 0.05 and OR = 1.52, 95% CI = 1.18–1.97, *P* = 0.004) and renovascular disease (respectively OR = 1.53, 95% CI = 1.10–2.11, *P* = 0.03 and OR = 1.86, 95% CI = 1.25–2.77, *P* = 0.002), however the estimated type II error ranged between 9–28%.

**Table 3:** Case-control study for the association between TLR single nucleotide polymorphsims and end-stage renal disease.

Gene	HGVS name	MAF cases (%)	MAF controls (%)	OR^1^	95% CI^1^	*P*-value^2^	Type II error when *P* < 0.05^3^
TLR1	p.His305Leu	9.5	2.6	4.79	2.35–9.75	0.0002	0%
TLR1	p.Asn248Ser	30.0	25.9	1.26	1.07–1.47	0.04	6%
TLR2	p.Arg753Gln	9.6	5.1	0.73	0.22–2.43	1	
TLR4	p.Asp299Gly	5.8	5.4	1.27	0.52–3.12	1	
TLR4	p.Thr399Ile	6.0	5.5	1.77	0.57–5.51	1	
TLR5	p.Arg392Ter	8.2	7.1	2.93	1.03–8.33	0.4	
TLR5	p.Phe616Leu	44.6	42.6	0.92	0.82–1.04	1	
TLR6	p.Ser249Pro	38.6	41.0	0.92	0.81–1.04	1	
TLR7	p.Gln11Leu	21.7	21.1	1.05	0.85–1.30	1	
TLR8	p.Met1Val	27.9	22.6	1.37	1.14–1.64	0.008	1%

^1^Per allele odds ratios (OR) and 95% confidence intervals (CI) based on additive genetic logistic regression models adjusted for age and gender, taking case-control relatedness into consideration (DFAM algorithm). HGVS = Human Genome Variation Society.

^2^ *P*-values are Bonferroni corrected.

^3^Estimates of the type II errors (100%–power) were calculated according to the methods by Skol *et al*. [23] with the data as mentioned in the table and an end-stage renal disease prevalence of 0.1% (estimate in The Netherlands).

**Table 4:** Case-control study for the association between TLR single nucleotide polymorphisms and end-stage renal disease per underlying disease category.

Gene	HGVS name	Renal disease	N cases	MAF cases(%)	MAF controls(%)	OR^1^	95% CI^1^	*P*-value^2^	Type II error when *P* < 0.05^3^
TLR1	p.His305Leu	Congenital	216	10.4	2.6	4.94	2.26–10.84	0.0002	0%
		Glomerulonephritis	299	8.4	2.6	4.91	2.31–10.41	0.0001	0%
		Pyelonephritis	126	11.5	2.6	6.71	3.10–14.53	<0.0001	0%
		Renovascular	102	9.8	2.6	4.37	1.83–10.43	0.003	0%
TLR1	p.Asn248Ser	Congenital	216	25.9	25.9	1.02	0.76–1.37	1	
		Glomerulonephritis	299	32.6	25.9	1.33	1.05–1.67	0.05	16%
		Pyelonephritis	126	27.0	25.9	1.27	0.94–1.71	0.4	
		Renovascular	102	33.3	25.9	1.53	1.10–2.11	0.03	28%
TLR8	p.Met1Val	Congenital	216	24.6	22.6	1.18	0.86–1.64	0.9	
		Glomerulonephritis	299	29.4	22.6	1.52	1.18–1.97	0.004	9%
		Pyelonephritis	126	28.1	22.6	1.24	0.85–1.81	0.8	
		Renovascular	102	33.6	22.6	1.86	1.25–2.77	0.002	17%

^1^Per allele odds ratios (OR) and 95% confidence intervals (CI) based on additive genetic logistic regression models adjusted for age and gender, taking case-control relatedness into consideration (DFAM algorithm). HGVS = Human Genome Variation Society.

^2^ *P*-values are Bonferroni corrected.

^3^ *Post hoc* estimates of the type II errors (100%–power) were calculated according to the methods by Skol *et al*. [23] with the data as mentioned in the table and an end-stage renal disease prevalence of 0.1% (estimate in The Netherlands).

### Role of TLR sequence variants in relation to delayed graft function

Delayed graft function (DGF) occurred in 328 of 1116 patients (29%). DGF was observed in 37% (317/867) of recipients receiving a graft from a deceased donor and 4% (11/249) in recipients of a living donor. We analysed the association of TLR SNPs with DGF in all included patients and separately in recipients of a deceased donor, since TLR activation is more prominently observed in the context of deceased donation [6]. In univariable analyses, where all donor and recipient SNPs were tested separately, none of the TLR SNPs associated with DGF after Bonferroni correction (Table 5). As expected, cold ischemia time (*P* < 0.0001), donor age (*P* = 0.0008) and recipient age (*P* = 0.0005) were significantly associated with the occurrence of DGF. The model that included these 3 parameters had an Akaike information criterion (AIC) of 1272.5. Since TLRs are highly redundant, we also tested the performance of a logistic regression model that included all TLR variants in one multivariable model. Adding all donor and recipient SNPs to the crude multivariable model of cold ischemia time, donor age and recipient age again resulted in a higher AIC (1289.4), showing no additional value for variants in TLR genes in explaining the occurrence of DGF. Similar results were obtained when only recipients of a deceased donor were analysed (S1 Table). We therefore conclude that variants in TLR genes are not associated with the occurrence of delayed graft function after transplantation.

**Table 5:** Association of TLR single nucleotide polymorphism with delayed graft function in univariable logistic regression analysis.

Gene	HGVS name	Allele combination^1^	Donor			Recipient		
			OR	95% CI	*P*-value^2^	OR	95% CI	*P*-value^2^
TLR1	p.His305Leu	T/a	0.69	0.34–1.30	1	1.12	0.75–1.67	1
		a/a	2.37	0.09–60.01	1	0.55	0.23–1.13	1
TLR1	p.Asn248Ser	C/t	0.86	0.65–1.13	1	0.85	0.64–1.12	1
		t/t	0.65	0.37–1.11	1	1.64	1.07–2.50	0.4
TLR2	p.Arg753Gln	G/a	0.71	0.44–1.11	1	0.74	0.52–1.04	1
		a/a	-	-	-	-	-	-
TLR4	p.Asp299Gly	A/g	1.30	0.86–1.94	1	0.82	0.53–1.24	1
		g/g	2.48	0.10–62.79	1	1.18	0.05–12.33	1
TLR4	p.Thr399Ile	C/t	1.28	0.85–1.90	1	0.85	0.56–1.28	1
		t/t	-	-	-	1.18	0.05–12.37	1
TLR5	p.Arg392Ter	G/a	1.35	0.94–1.93	1	1.08	0.75–1.54	1
		a/a	-	-	-	1.46	0.30–6.00	1
TLR5	p.Phe616Leu	A/g	0.92	0.69–1.24	1	0.78	0.59–1.05	1
		g/g	1.15	0.80–1.67	1	0.88	0.60–1.28	1
TLR6	p.Ser249Pro	G/a	1.00	0.75–1.33	1	0.91	0.68–1.20	1
		a/a	0.97	0.66–1.41	1	1.01	0.69–1.46	1
TLR7	p.Gln11Leu	A/t	0.88	0.63–1.23	1	0.83	0.57–1.19	1
		t/t	1.25	0.83–1.85	1	0.74	0.48–1.09	1
TLR8	p.Met1Val	A/g	0.96	0.68–1.34	1	1.09	0.77–1.55	1
		g/g	1.04	0.70–1.53	1	1.12	0.80–1.54	1

OR = odds ratio (^1^per allele combination as compared to the homozygous dominant allele combination)

CI = confidence interval, HGVS = Human Genome Variation Society. The results represent univariable crude models, i.e. no other independent variables were included.

^2^ *P*-values are Bonferroni corrected.

### Role of TLR sequence variants in relation to acute rejection

The median time of freedom-of-rejection was 51 months (interquartile range 1–105 months). The overall cumulative incidence of biopsy-proven acute rejection (BPAR) after renal transplantation was 34% (378/1116). In univariable analyses, where all donor and recipient SNPs were tested separately, none of the SNPs in the TLR genes associated with BPAR after Bonferroni correction (Table 6). In our cohort, most of the variation in the occurrence of BPAR could be explained by a preceding period of DGF (*P* = 0.01), recipient age (*P* < 0.0001) and the number of HLA mismatches (*P* < 0.0001), which resulted in an AIC of 4125.6. When we added all donor and recipient TLR variants to the model that included DGF, recipient age and number of HLA mismatches, again a higher AIC was calculated (AIC = 4142.0), indicating no additional explanatory value by the TLR variants for the cumulative incidence of BPAR.

**Table 6:** Association of TLR single nucleotide polymorphism with biopsy-proven acute rejection.

Gene	HGVS name	Allele combination^1^	Donor			Recipient		
			HR	95% CI	*P*-value^2^	HR	95% CI	*P*-value^2^
TLR1	p.His305Leu	T/a	0.68	0.39–1.18	1	1.60	1.20–2.12	0.1
		a/a	1.86	0.26–13.21		1.02	0.60–1.74	
TLR1	p.Asn248Ser	C/t	0.97	0.78–1.20	1	1.09	0.88–1.35	1
		t/t	1.37	0.95–1.98		1.04	0.72–1.48	
TLR2	p.Arg753Gln	G/a	0.84	0.59–1.20	1	1.32	1.04–1.68	0.9
		a/a	-	-		2.92	0.41–20.84	
TLR4	p.Asp299Gly	A/g	0.85	0.60–1.20	1	0.90	0.65–1.25	1
		g/g	-	-		1.05	0.15–7.49	
TLR4	p.Thr399Ile	C/t	0.91	0.65–1.27	1	0.94	0.68–1.30	1
		t/t	-	-		1.06	0.15–7.53	
TLR5	p.Arg392Ter	G/a	1.00	0.75–1.34	0.3	0.80	0.59–1.09	1
		a/a	10.52	1.47–75.19		1.04	0.33–3.24	
TLR5	p.Phe616Leu	A/g	1.08	0.85–1.36	1	0.92	0.74–1.16	1
		g/g	1.03	0.76–1.38		0.97	0.72–1.30	
TLR6	p.Ser249Pro	G/a	0.89	0.72–1.12	1	0.73	0.59–0.92	0.1
		a/a	0.86	0.64–1.16		1.06	0.81–1.40	
TLR7	p.Gln11Leu	A/t	0.99	0.76–1.28	1	0.91	0.68–1.22	1
		t/t	1.25	0.92–1.28		1.17	0.88–1.56	
TLR8	p.Met1Val	A/g	1.25	0.98–1.61	1	1.07	0.81–1.41	1
		g/g	0.96	0.70–1.32		1.20	0.93–1.54	

HR = hazard ratio (^**1**^per allele combination as compared to the homozygous dominant allele combination)

CI = confidence interval, HGVS = Human Genome Variation Society. The results represent univariable crude models, i.e. no other independent variables were included.

^2^ *P*-values are calculated by log rank tests after Bonferroni correction for multiple comparisons.

### Role of TLR sequence variants in relation to graft failure

Median overall graft survival was 5.5 years (interquartile range 2.9–8.9 years). The overall cumulative incidence of death-censored graft failure was 191/1116 (17%) of which 124/215 (66%, 11% of total) failed due to rejection. In line with our negative findings concerning the lack of association of the TLR variants with the surrogate endpoints DGF and BPAR, none of the variants associated with the cumulative incidence of death-censored graft failure (Table 7). In our cohort, patients that underwent an episode of BPAR had worse death-censored graft survival (*P* < 0.0001, AIC = 2504.3). Again, a multivariable model that included all donor and recipient TLR variants plus the occurrence of an episode of BPAR did not improve the goodness-of-fit of the model (AIC = 2530.2), therefore indicating no additional value for the TLR variants in explaining the development of death-censored graft failure after transplantation.

**Table 7:** Association of TLR single nucleotide polymorphism with death-censored graft survival.

Gene	HGVS name	Allele combination^1^	Donor			Recipient		
			HR	95% CI	*P*-value^2^	HR	95% CI	*P*-value^2^
TLR1	p.His305Leu	T/a	1.02	0.52–2.00	1	1.01	0.65–1.56	1
		a/a	-	-		0.56	0.23–1.37	
TLR1	p.Asn248Ser	C/t	1.13	0.84–1.52	1	1.11	0.82–1.50	1
		t/t	0.93	0.51–1.69		1.44	0.92–2.24	
TLR2	p.Arg753Gln	G/a	1.13	0.72–1.77	1	1.29	0.93–1.80	1
		a/a	-	-		-	-	
TLR4	p.Asp299Gly	A/g	1.15	0.74–1.80	1	0.80	0.50–1.29	1
		g/g	-	-		-	-	
TLR4	p.Thr399Ile	C/t	1.17	0.76–1.81	1	0.74	0.45–1.20	1
		t/t	-	-		-	-	
TLR5	p.Arg392Ter	G/a	1.31	0.90–1.90	1	0.84	0.55–1.27	1
		a/a	-	-		0.77	0.11–5.52	
TLR5	p.Phe616Leu	A/g	1.03	0.75–1.43	1	1.20	0.87–1.64	1
		g/g	0.82	0.53–1.28		0.70	0.44–1.13	
TLR6	p.Ser249Pro	G/a	1.19	0.87–1.64	1	0.85	0.62–1.17	1
		a/a	0.96	0.62–1.48		1.08	0.73–1.61	
TLR7	p.Gln11Leu	A/t	0.83	0.57–1.19	1	0.89	0.59–1.35	1
		t/t	0.96	0.61–1.50		1.41	0.96–2.06	
TLR8	p.Met1Val	A/g	0.99	0.68–1.44	1	1.05	0.71–1.56	1
		g/g	1.20	0.80–1.80		1.28	0.91–1.82	

HR = hazard ratio (^1^per allele combination as compared to the homozygous dominant allele combination)

CI = confidence interval, HGVS = Human Genome Variation Society. The results represent univariable crude models, i.e. no other independent variables were included.

^2^ *P*-values are calculated by log rank tests after Bonferroni correction for multiple comparisons.

## Discussion

TLR-signaling and control by their negative regulators is of special interest in renal diseases because of their expression pattern in murine and human kidneys and the role they play in experimental models of acute and chronic renal injury [46–55]. Contribution of other TLRs (besides the TLR2-4-6 axis) on progression to pre-transplant ESRD and renal outcome after transplantation is a relatively unexplored field. In this large cohort of renal transplant recipients, we observed that 1) TLR1 p.His305Leu, TLR1 p.Asn248Ser and TLR8 p.Met1Val significantly associated with the prevalence of end-stage renal disease, and 2) SNPs in TLR genes do not explain the occurrence of delayed graft function, biopsy-proven acute rejection and subsequently death-censored graft failure after transplantation.

Thus far, relatively few studies have investigated the contribution of non-synonymous polymorphisms in TLR-related genes and their association with renal diseases of the native kidneys and the development of chronic kidney disease (CKD) [12,56–62]. Table 8 summarizes the studies that we could identify from the literature in which the same TLR variants were analyzed as in our study. Lee *et al*. described a higher frequency of the minor allele for TLR1 p.Asn248Ser in pediatric patients with IgA nephropathy as compared to healthy controls [56], which might be compared to the higher frequency of the minor allele for this variant in patients with immunecomplex-mediated glomerulonephritis in our cohort. Unfortunately, in this study TLR1 p.His305Leu, the variant that showed a robust and very relevant association with end-stage renal disease in our cohort, was not investigated. TLR4 p.Asp299Gly and p.Thr399Ile are by far the most studied TLR variants in the literature [12,58–62]. Only the study by Akil *et al*. found a significant association between the minor allele frequency of any of the two TLR4 variants and the prevalence of chronic kidney disease [60]. The vast majority of studies however did not find an association, which is in line with our study and because p.Asp299Gly and p.Thr399Ile are in high linkage disequilibrium, one would expect a similar effect for both variants in this case. Interestingly, two [60,61] of three studies [60–62] found an association of the minor allele frequency for the TLR4 variant p.Asp299Gly with pyelonephritis (both with and without secondary chronic kidney disease), which could unfortunately not be investigated in such detail in our study. In line with our study, Cheng *et al*. did not find an association of the minor allele of TLR5 variant p.Arg392Ter with native kidney diseases [57]. The residual TLR variants, which includes TLR8 p.Met1Val that showed an association with end-stage renal disease in our cohort, have not been described before. Due to the lack of functional TLR pathway testing in patients carrying these TLR SNPs associated with end-stage renal disease, we consider our study hypothesis-generating.

**Table 8:** Association of the TLR gene polymorphisms with renal outcomes as described in the literature.

Gene	HGVS name	Author, year	Reference	Country	Native / Transplant	Disease	Controls	Effect of the minor allele
TLR1	p.His305Leu	-	-	-	-	-	-	-
TLR1	p.Asn248Ser	Lee, 2011	[56]	Korean	Native	GN	Healthy	***Increased risk***
		Cheng, 2013	[57]	Taiwan	Native	PN	no PN	No effect
TLR2	p.Arg753Gln	Mutlubas, 2009	[58]	Turkey	Native	CKD	Healthy	***Increased risk***
		Soylu, 2010	[59]	Turkey	Native	GN	no GN	No effect
		Krüger, 2010	[13]	Germany	Transplant	DGF	no DGF	No effect
		Krüger, 2010	[13]	Germany	Transplant	AR	no AR	No effect
		Mutlubas, 2009	[58]	Turkey	Transplant	GF	no GF	No effect
		Krüger, 2010	[13]	Germany	Transplant	GF	no GF	No effect
TLR4	p.Asp299Gly	Nogueira, 2007	[12]	Brazil	Native	CKD	Healthy	No effect
		Mutlubas, 2009	[58]	Turkey	Native	CKD	Healthy	No effect
		Akil, 2012	[60]	Turkey	Native	CKD	no CKD	***Increased risk***
		Bayram, 2013	[62]	Turkey	Native	CKD	no CKD	No effect
		Soylu, 2010	[59]	Turkey	Native	GN	no GN	No effect
		Karoly, 2007	[61]	Hungary	Native	PN	no PN	***Increased risk***
		Akil, 2012	[60]	Turkey	Native	PN	no PN	***Increased risk***
		Bayram, 2013	[62]	Turkey	Native	PN	no PN	No effect
		Nogueira, 2007	[12]	Brazil	Transplant	DGF^2^	no DGF^2^	No effect
		Krüger, 2010	[13]	Germany	Transplant	DGF^2^	no DGF^2^	No effect
		Ducloux, 2005	[7]	France	Transplant	AR^2^	no AR^2^	***Decreased risk***
		Palmer, 2006	[8]	United States	Transplant	AR^1^	no AR^1^	***Decreased risk***
		Fekete, 2006	[63]	Hungary	Transplant	AR^2^	no AR^2^	***Decreased risk***
		Nogueira, 2007	[12]	Brazil	Transplant	AR^2^	no AR^2^	No effect
		Krüger, 2010	[13]	Germany	Transplant	AR^2^	no AR^2^	No effect
		Krichen, 2013	[18]	Tunesia	Transplant	AR^2^	no AR^2^	No effect
		Nogueira, 2007	[12]	Brazil	Transplant	PN^2^	no PN^2^	No effect
		Ducloux, 2005	[7]	France	Transplant	GF^2^	no GF^2^	No effect
		Mutlubas, 2009	[58]	Turkey	Transplant	GF^2^	no GF^2^	No effect
		Krüger, 2010	[13]	Germany	Transplant	GF^2^	no GF^2^	No effect
TLR4	p.Thr399Ile	Nogueira, 2007	[12]	Brazil	Native	CKD	Healthy	No effect
		Mutlubas, 2009	[58]	Turkey	Native	CKD	Healthy	No effect
		Bayram, 2013	[62]	Turkey	Native	CKD	no CKD	No effect
		Soylu, 2010	[59]	Turkey	Native	GN	no GN	No effect
		Nogueira, 2007	[12]	Brazil	Transplant	DGF	no DGF^2^	No effect
		Krüger, 2010	[13]	Germany	Transplant	DGF	no DGF^2^	No effect
		Ducloux, 2005	[7]	France	Transplant	AR^2^	no AR^2^	***Decreased risk***
		Palmer, 2006	[8]	United States	Transplant	AR^1^	no AR^1^	***Decreased risk***
		Nogueira, 2007	[12]	Brazil	Transplant	AR^2^	no AR^2^	No effect
		Krüger, 2010	[13]	Germany	Transplant	AR^2^	no AR^2^	No effect
		Nogueira, 2007	[12]	Brazil	Transplant	PN^2^	no PN^2^	No effect
		Ducloux, 2005	[7]	France	Transplant	GF^2^	no GF^2^	No effect
		Mutlubas, 2009	[58]	Turkey	Transplant	GF^2^	no GF^2^	No effect
		Krüger, 2010	[13]	Germany	Transplant	GF^2^	no GF^2^	No effect
TLR5	p.Arg392Ter	Cheng, 2013	[57]	Taiwan	Native	PN	no PN	No effect
		Krüger, 2010	[13]	Germany	Transplant	DGF	no DGF	No effect
		Krüger, 2010	[13]	Germany	Transplant	AR	no AR	No effect
		Krüger, 2010	[13]	Germany	Transplant	GF	no GF	No effect
TLR5	p.Phe616Leu	-	-	-	-	-	-	-
TLR6	p.Ser249Pro	-	-	-	-	-	-	-
TLR7	p.Gln11Leu	-	-	-	-	-	-	-
TLR8	p.Met1Val	-	-	-	-	-	-	-

^1^SNP in donors

^2^SNP in recipients.

Native = renal diseases of the native kidneys, transplant = renal diseases after transplantation, GN = glomerulonephritis, Healthy = healthy controls, PN = pyelonephritis, CKD = chronic kidney disease, DGF = delayed graft function, AR = acute rejection, GF = graft failure.

Chronic renal allograft failure on the other hand is known to develop from both immune and non-immune damage to the graft. In our study we were unable to show an association of any of the TLR SNPs in either donor or recipient with surrogate and definite outcomes. Table 8 provides a list of the same TLR SNPs and their association with transplant outcomes as described in the literature [7,8,12,13,18,58,63]. Comparable to our study, Krüger *et al*. [13] and Mutlubas *et al*. [58] did not identify an association of TLR2 p.Arg753Gln with renal transplant outcomes. In the context of renal transplantation, TLR4 p.Asp299Gly and p.Thr399Ile are also among the most studied TLR SNPs [7,8,12,13,18,58,63]. Contrary to the well-known role for TLR4 in experimental ischemia-reperfusion injury, none of the studies (including the current) found an association with either of the two TLR4 SNPs with the development of delayed graft function [12,13]. Delayed graft function is a heterogeneous and arbitrary outcome measure, that includes the effect of donation type, donor/recipient age, ischemia times (cold and warm) but also allo-immune phenomena, which makes a direct comparison with experimentally controlled ischemia-reperfusion injury difficult and this difference should be taken into account when interpreting genetic analyses. A potential role for TLR4 SNPs in the context of acute rejection generated conflicting results with half of the studies describing a protective effect [7,8,63] and half of the studies describing no effect at all [12,13,18]. Our study, which is the largest investigating both TLR4 SNPs to date, is in line with the latter studies. Interestingly, none of the studies (including the current) describes an effect on the cumulative incidence of graft failure [7,13,58]. One could wonder why a loss-of-function polymorphism in TLR4 leads to a lower incidence of acute rejection, but not graft failure. If there is a role for TLR4 polymorphisms in renal transplantation, it is probably a minor one that is overshadowed by other immune and non-immune phenomena that take place in the graft. Finally, Krüger *et al*. [13], in line with our study, did not find an association of TLR5 p.Arg392Ter with renal transplant outcomes. Even though experimental renal transplantation studies have consistently shown that TLR engagement can break allograft tolerance while inhibition of for instance TLR2 and -4 signalling improves allograft acceptance [48,53,64–66], in human renal transplantation the consequences of non-synonymous SNPs in TLR genes appear to be not as devastating for transplant outcome.

In conclusion, TLR gene polymorphisms are enriched in patients with end-stage renal disease and may contribute to the final common pathway of renal injury whereas after renal transplantation, this effect for the TLR gene polymorphisms was not observed. This difference in effect size by the TLR gene polymorphisms highlights that the development of chronic kidney disease of the native kidneys and chronic kidney disease in the context of renal transplantation might be explained by different risk factors.

## Supporting Information

S1 Table Association of TLR single nucleotide polymorphism with delayed graft function in univariable logistic regression analysis in recipients of a deceased donor. (DOCX)
