# Supplementary material for: Toll-Like Receptor Family Polymorphisms Are Associated with Primary Renal Diseases but Not with Renal Outcomes Following Kidney Transplantation
Source: PLoS One. 2015 Oct 7;10(10):e0139769. doi: 10.1371/journal.pone.0139769 (PMC4596574; doi:10.1371/journal.pone.0139769)
Supplement: S1 Table — (DOCX) [file pone.0139769.s001.docx]

**S1 Table. Association of TLR single nucleotide polymorphism with delayed graft function in univariable logistic regression analysis in recipients of a deceased donor.**

| **Gene** | **HGVS name** | **Allele combination^1^** | **Donor** | | | **Recipient** | | |
| --- | --- | --- | --- | --- | --- | --- | --- | --- |
|  |  |  | **OR** | **95% CI** | ***P*-value^2^** | **OR** | **95% CI** | ***P*-value^2^** |
| TLR1 | p.His305Leu | T/a | 0.76 | 0.35 – 1.52 | 1 | 1.16 | 0.75 – 1.79 | 1 |
|  |  | a/a | 1.72 | 0.07 – 43.55 | 1 | 0.19 | 0.19 – 0.96 | 1 |
| TLR1 | p.Asn248Ser | C/t | 0.86 | 0.64 – 1.15 | 1 | 0.93 | 0.69 – 1.25 | 1 |
|  |  | t/t | 0.76 | 0.41 – 1.36 | 1 | 1.71 | 1.09 – 2.70 | 0.4 |
| TLR2 | p.Arg753Gln | G/a | 0.67 | 0.41 – 1.06 | 1 | 0.67 | 0.46 – 0.96 | 0.7 |
|  |  | a/a | - | - | - | - | - | - |
| TLR4 | p.Asp299Gly | A/g | 1.54 | 0.98 – 2.42 | 1 | 0.74 | 0.46 – 1.15 | 1 |
|  |  | g/g | - | - | 1 | - | - | 1 |
| TLR4 | p.Thr399Ile | C/t | 1.47 | 0.93 – 2.29 | 1 | 0.74 | 0.47 – 1.15 | 1 |
|  |  | t/t | - | - | - | - | - | 1 |
| TLR5 | p.Arg392Ter | G/a | 1.53 | 1.03 – 2.26 | 0.6 | 1.06 | 0.72 – 1.56 | 1 |
|  |  | a/a | - | - | - | 1.76 | 0.32 – 9.56 | 1 |
| TLR5 | p.Phe616Leu | A/g | 0.76 | 0.53 – 1.09 | 1 | 0.87 | 0.59 – 1.29 | 1 |
|  |  | g/g | 0.89 | 0.60 – 1.32 | 1 | 1.34 | 0.89 – 2.03 | 1 |
| TLR6 | p.Ser249Pro | G/a | 1.01 | 0.74 – 1.37 | 1 | 0.87 | 0.64 – 1.17 | 1 |
|  |  | a/a | 0.90 | 0.60 – 1.35 | 1 | 0.96 | 0.64 – 1.42 | 1 |
| TLR7 | p.Gln11Leu | A/t | 0.82 | 0.57 – 1.16 | 1 | 0.93 | 0.62 – 1.37 | 1 |
|  |  | t/t | 1.15 | 0.74 – 1.77 | 1 | 0.70 | 0.45 – 1.07 | 1 |
| TLR8 | p.Met1Val | A/g | 0.98 | 0.59 – 1.64 | 1 | 0.97 | 0.61 – 1.53 | 1 |
|  |  | g/g | 1.03 | 0.68 – 1.59 | 1 | 0.85 | 0.59 – 1.22 | 1 |

OR = odds ratio (^1^per allele combination as compared to the homozygous dominant allele combination), CI = confidence interval, HGVS = Human Genome Variation Society. The results represent univariable crude models, i.e. no other independent variables were included.

^2^*P*-values are Bonferroni corrected.
